# Everyone deserves a second chance: The importance of second opinions for left ventricular assist device candidacy

**DOI:** 10.1016/j.jhlto.2026.100531

**Published:** 2026-03-02

**Authors:** Timothy J. George, David A. Rawitscher, Nitin Kabra, Greg Milligan, Akash Rusia, J. Michael DiMaio, Haider Nazeer, Marika Harada, Aasim Afzal

**Affiliations:** Baylor Scott and White, The Heart Hospital, Plano, TX

**Keywords:** Left ventricular assist device, LVAD, Selection, Risk stratification, Second opinion

## Abstract

**Introduction:**

Although left ventricular assist device (LVAD) therapy improves survival and quality of life in patients with end-stage heart failure, appropriate patient selection is complex, with many patients being turned down for implantation because of perceived medical, surgical, or social barriers. However, some patients turned down for an implant at one center will go on to be successfully implanted elsewhere.

**Methods:**

We conducted a retrospective review of all primary LVAD implantations at our center. Primary stratification was by prior turndown status. Primary outcome was survival as assessed by the Kaplan-Meier method and Cox proportional hazards regression modeling. Secondary outcomes included length of stay and commonly encountered postoperative complications.

**Results:**

From 2017 to 2025, 237 patients underwent primary LVAD implantation, 28 (11.81%) of whom were turned down for implantation at another center. When stratified by turndown status, there was no difference in operative mortality (17/209, 8.13% vs 2/28, 7.14%, *p* = 0.86) or 5-year survival (*p* = 0.73). On multivariable analysis, prior LVAD turndown was not associated with 1-year survival (HR: 0.41[0.09-1.17], *p* = 0.23). Moreover, patients previously turned down for LVAD had similar lengths of stay and rates of bleeding, stroke, respiratory failure, renal failure, and right ventricular failure.

**Conclusions:**

Patients turned down for LVAD implantation at another center prior to referral to our center had similar short-term morbidity and mortality compared to patients not previously turned down for surgery. These findings suggest that patients with end-stage heart failure who are turned down for LVAD therapy at one center should seek or be referred for a second opinion at another center.

## Background

Advanced heart failure remains a pervasive problem, contributing to substantial morbidity, mortality, and healthcare costs.^1^, ^2^ Patients with end-stage heart failure have 3 options: heart transplantation, left ventricular assist device (LVAD) implantation, and palliative care.^3^ LVAD therapy has emerged as an important option in patients with end-stage heart failure both as a bridge to transplantation and as destination therapy.^4^, ^5^ Multiple studies have demonstrated the efficacy of LVAD therapy, resulting in superior survival and improved quality of life compared to optimal medical therapy alone.^1^, ^4^, ^6^, ^7^, ^8^

Despite the success of LVAD therapy, multidisciplinary patient selection involving medical, surgical, and social factors is critical for both short and long-term success.^9^, ^10^ Patient factors including surgical complexity, medical co-morbidities, and psychosocial issues can all contribute to increased early and late mortality, thus potentially leading to LVAD therapy turndowns at implanting centers.^11^, ^12^, ^13^, ^14^ However, despite multiple attempts to standardize and codify surgical, medical, and social risk with various algorithms and calculators, risk assessment remains ultimately subjective. Moreover, each implanting center may have a different risk tolerance based on either patient or programmatic factors, leading to inconsistencies in care. Thus, a patient who is not a candidate at one center or a borderline candidate at another center may be an acceptable or even “on target” candidate at a different hospital.

Second opinions in advanced heart failure management are increasingly recognized as important and valuable, with recent guidelines emphasizing timely referral to specialized and high-volume centers.^15^, ^16^ Despite these guidelines, limited data exist on outcomes of patients turned down at 1 center who are subsequently implanted at another center.^12^, ^17^ Therefore, we undertook this study to evaluate whether patients previously declined LVAD implantation at other centers experience similar outcomes to those without a prior turndown. We hypothesized that careful selection at our high-volume, destination therapy center might yield comparable outcomes regardless of prior LVAD denial.

## Methods

We conducted a retrospective review of all primary LVAD implantations performed at Baylor Scott and White, The Heart Hospital, Plano, from 2017 to 2025. Patients with a prior LVAD implantation who underwent exchange were excluded. The study was approved by The Baylor Scott and White Institutional Review Board, and informed consent was waived. During the period of study, the Heartmate 3 LVAD device (Abbott Laboratories, Chicago, IL) was used exclusively.

As a destination therapy center, all patients implanted with an LVAD were either formally declined for heart transplantation by a transplant center, determined not to be a heart transplant candidate by an advanced heart failure cardiologist with transplant privileges, or personally chose LVAD over transplant for a net prolongation of life strategy or other personal reasons. However, this initial ineligibility for transplant does not constitute a “turndown” as defined in this study. Rather, for the present study, patients were stratified into 2 groups: patients turned down by another LVAD center for LVAD implantation specifically (not simply turned down for transplant but also turned down for LVAD) and those not formally declined for LVAD at another program (these patients were turned down for transplant). In this study, all of the turndown patients were implanted on the same admission; thus, none of the perceived barriers at another center were able to be ameliorated prior to implant.

Baseline demographics, laboratory values, measures of acuity, Intermacs profiles, operative data, survival status, and complications were extracted from the electronic medical record. In attempting to determine why patients were declined for LVAD at other centers, we have broadly classified patients into 3 groups: those declined for surgical risk, those declined for medical risk, and those declined for psychosocial risk. Since every center evaluates, determines, and stratifies risk differently, these are just broad categorizations based on the referring center’s own criteria and are based on self-reporting.

For simplicity, in defining commonly encountered complications, we used the most concrete endpoints possible. Therefore, for renal failure, we measured renal failure requiring any renal replacement therapy (intermittent hemodialysis or continuous renal replacement therapy) for volume removal, clearance, or both. For respiratory failure, we used the need for tracheostomy as the end point. Similarly, for right ventricular failure, we used the need for temporary right ventricular mechanical circulatory support (postoperative ECMO or RVAD—Protek or Impella RP). Patients requiring prolonged inotropes only were not included.

Continuous parametric data are presented as means with standard deviations, and these comparisons were made using the Student’s *t*-test. Continuous non-parametric data are presented as medians with accompanying interquartile ranges and were compared using the Kruskal-Wallis test. Categorical variables are presented as whole numbers with percentages and were compared using either the Chi-squared test or Fisher’s exact test as appropriate. Survival was estimated using the Kaplan-Meier method, and survival comparisons were performed using the log-rank test.

Multivariable Cox proportional hazards regression models were constructed to further analyze 1-year mortality. To construct our model, preoperative variables were initially evaluated in univariate fashion. Variables with a univariate *p*-value < 0.2, variables with biologic plausibility, and variables with prior literature support were then incorporated into the model in forward and backward stepwise nested fashion using likelihood ratio testing to build the most parsimonious model.

For all statistical tests, 2-tailed *p* < 0.05 was considered statistically significant. Statistical analysis was performed using Stata/BE 19.5 (StataCorp, College Station, Texas).

## Results

From 2017 to 2025, 237 patients underwent primary LVAD implantation. The average age was 62.08 ± 12.06 years, 191 (80.59%) were male; the mean creatinine was 1.42 ± 0.54 mg/dL, and 88 (37.13%) were Intermacs profile 1. 60 (25.32%) patients required a reoperative sternotomy, and 90 (37.97%) required a concomitant valve operation with an average cardiopulmonary bypass time of 98.56 ± 38.25 minutes.

**Figure 1:**
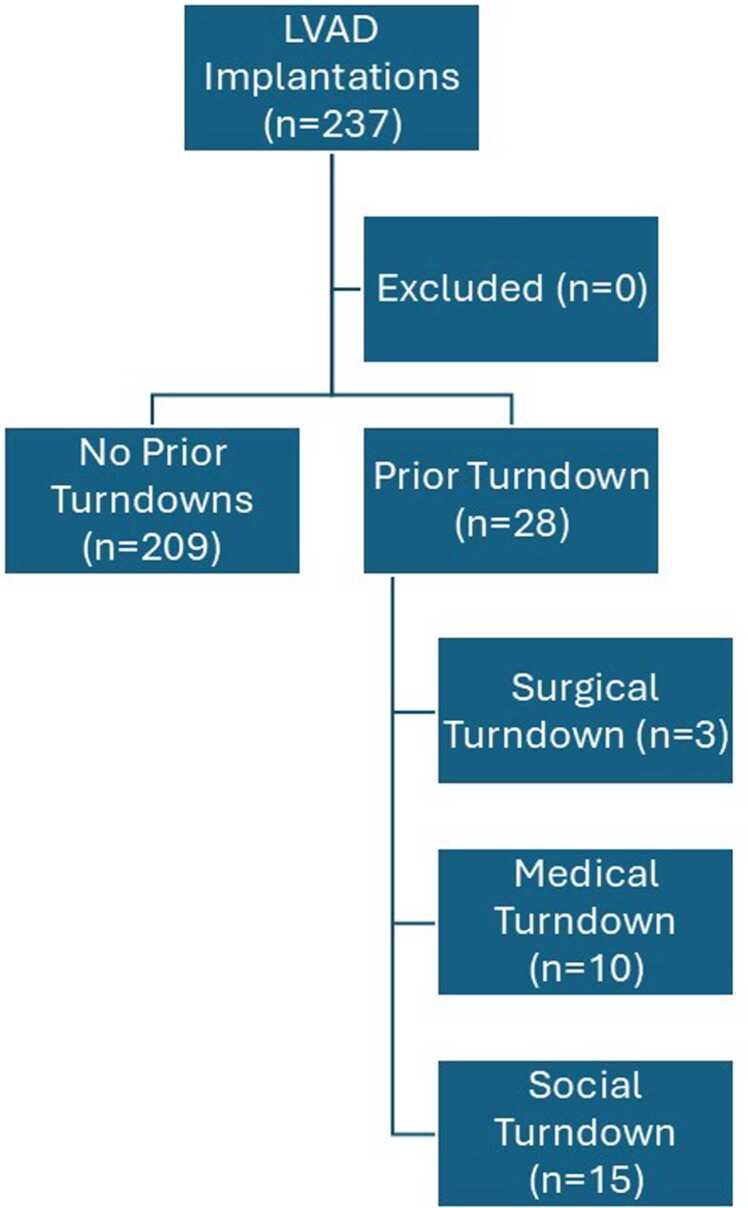
CONSORT diagram of study design.

Of the 237 patients, 28 (11.81%) were turned down for LVAD implantation at another LVAD center (Figure 1). Reasons for turndown are subjective and sometimes multi-faceted but broadly fit into 3 categories: surgical complexity, medical co-morbidities, and social issues (as defined by the referring center). Of the 28 turndowns, 3 (10.7%) were declined for surgical complexity, 10 (35.71%) were declined for medical co-morbidities, and 15 (53.57%) were declined for social reasons. When stratified by prior LVAD implantation turndown, patients who were previously turned down at other centers but offered LVAD at our center tended to be younger (62.92 ± 11.71 vs 55.75 ± 12.99 years, *p* = 0.01) and were more likely to be Black (37/209, 17.70% vs 13/28, 46.43%, *p* < 0.01; Table 1). Other demographics, laboratory values, measures of acuity, and operative variables were similar between the 2 groups.

**Table 1:** Baseline Characteristics Stratified by Prior LVAD Turndown

Variable	No prior turndown (*n* = 209)	Prior turndown (*n* = 28)	*p*-value
*Demographics*			
Age (years)	62.92±11.71	55.75±12.99	0.01
Male gender (*n*, %)	170 (81.34%)	21 (75.00%)	0.43
White race (*n*, %)	149 (71.29%)	11 (39.29%)	
Black race (*n*, %)	37 (17.70%)	13 (46.43%)	
Hispanic race (*n*, %)	19 (9.09%)	2 (7.14%)	<0.01
Ischemic etiology (*n*, %)	98 (53.11%)	19 (67.86%)	0.14
Body mass index (mg/kg^2^)	28.28±6.49	27.90±7.06	0.77
*Laboratory values*			
Creatinine (mg/dL)	1.43±0.54	1.37±0.57	0.62
Total bilirubin (mg/dL)	0.90 [0.6-1.4]	0.95 [0.55-1.55]	0.99
Sodium (meq/L)	135.23±4.33	134.50±3.50	0.39
Hemoglobin (g/dL)	11.71±8.71	10.04±1.98	0.31
*Measures of acuity*			
Intermacs Profile 1 (*n*, %)	75 (35.89%)	13 (46.43%)	
Intermacs Profile 2 (*n*, %)	56 (26.79%)	10 (35.71%)	
Intermacs Profile 3 (*n*, %)	64 (30.62%)	5 (17.86%)	
Intermacs Profile 4 (*n*, %)	14 (6.70%)	0 (0.00%)	0.20
Need for Impella support (*n*, %)	73 (34.93%)	12 (42.86%)	041
*Operative variables*			
Reoperative surgery (*n*, %)	55 (26.32%)	5 (17.86%)	0.33
Concomitant surgery (*n*, %)	79 (37.80%)	11 (39.29%)	0.88
CPB time (minutes)	97.52±35.92	106.21±52.46	0.26
Need for cardiac arrest (*n*, %)	24 (11.48%)	2 (7.14%)	0.49

Abbreviations: *n*, number; mg, milligram; kg, kilogram; mL, milliliter; min, minute, m^2^, meters squared; meq, milliequivalents; L, liter; %, percent.

**Figure 2:**
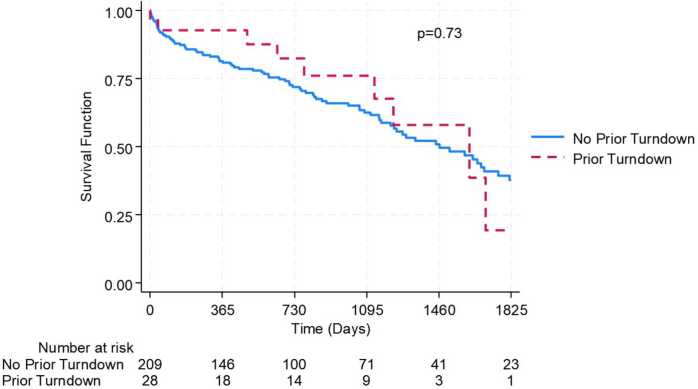
5-Year Kaplan-Meier survival stratified by prior LVAD implantation turndown.

In the overall cohort, operative mortality was 8.02% (19 patients) with a 1-year survival of 83.97%. Kaplan-Meier estimates of survival at 1, 2, 3, and 4 years were 82.64%, 73.02%, 63.91%, and 51.48%, respectively. When stratified by prior turndown status, there was no difference in operative mortality (17/209, 8.13% vs 2/28, 7.14%, *p* = 0.86) or short-term survival: 1-year (81.38% vs 92.72%, *p* = 0.20); 2-year (71.88% vs 82.42%, *p* = 0.28); and 3-year (62.51% vs 76.08%, *p* = 0.27; Figure 2). While the numbers get smaller, survival was still similar at 5 years (*p* = 0.73). Although numerically small, the operative mortality for patients turned down at other centers for either medical or surgical risk was 7.69% with a 1-year survival of 92.31%, while the operative mortality for patients turned down at other centers for social reasons was 6.67% with a 1-year survival of 93.33%. On multivariable analysis, being turned down for LVAD implantation at another center was not associated with an increased risk of 1-year mortality (HR: 0.41 [0.09-1.17], *p* = 0.23; Table 2). In fact, there was a mild signal that being turned down at another center might be protective, though this did not reach statistical significance. Elevated preoperative creatinine was associated with mortality (HR: 1.94[1.15-3.29], *p* = 0.01).

**Table 2:** Multivariable Cox Proportional Hazards Regression Model of 1-Year Mortality

Variable	Hazard ratio	95% CI	*p*-value
Prior turn down	0.41	[0.09-1.77]	0.23
Age (per year)	1.02	[0.99-1.06]	0.15
Creatinine (per mg/dL)	1.94	[1.15-3.29]	0.01
Total bilirubin (per mg/dL)	0.94	[0.66-1.34]	0.72
Hemoglobin (g/dL)	0.99	[0.95-1.04]	0.90
Need for preoperative impella	1.39	[0.68-2.84]	0.37
CPB time (per minute)	1.00	[0.99-1.01]	0.24

Abbreviations: mg, milligrams; dL, deciliters; g, grams; CPB, cardiopulmonary bypass; CI, confidence interval.

Patients turned down for LVAD at other centers had similar preoperative length of stay (10.09 ± 6.53 vs 9.71 ± 5.11 days, *p* = 0.76), similar postoperative length of stay (20.91 ± 12.19 vs 24.54 ± 14.59 days, *p* = 0.15), and similar overall length of stay (31.13 ± 14.96 vs 34.32 ± 16.70 days, *p* = 0.30). These patients also had a similar rate of reoperation for bleeding (44/209, 21.05% vs 6/28 21.43%, *p* = 0.96), a similar rate of perioperative stroke (11/209, 5.26% vs 0/28, 0.00%, *p* = 0.21), a similar rate of respiratory failure requiring tracheostomy (34/209, 16.27% vs 4/28, 14.29%, *p* = 0.79), and a similar rate of renal failure requiring at least temporary renal replacement therapy (40/209, 19.14% vs 5/28, 17.86%, *p* = 0.87). Although not statistically significant, patients turned down for LVAD implantation at other centers demonstrated a higher tendency for right heart failure requiring temporary right ventricular assist device placement (39/209, 18.66% vs 9/28, 32.14%, *p* = 0.09).

## Discussion

In this retrospective analysis of 237 primary LVAD implantations at a single center, we found no significant differences in operative mortality or short-term survival in patients previously turned down for LVAD therapy at other centers. After adjusting for other preoperative variables, these results were confirmed on multivariable analysis. On multivariable analysis, preoperative renal dysfunction was strongly associated with mortality. Additionally, there were no differences in major complications, including bleeding, stroke, renal failure, respiratory failure, or right ventricular failure. Moreover, these patients had similar pre- and post-operative lengths of stay. These findings suggest that some patients turned down for LVAD implantation at one center may benefit from implantation at a different center, underscoring the importance of a second opinion.

The early results in our study are similar to those reported in the STS database at 1 and 2 years for continuous flow devices. Although the longer-term results demonstrate lower survival compared to the full STS cohort, they compare favorably to the DT cohort.^18^ Several additional findings are noteworthy. First, the patients with prior turndown tended to be younger and were more likely to be Black and thus less likely to be White. In regards to age, we would speculate that we have a higher surgical and medical risk tolerance for younger patients than older patients, biasing our sample toward youth. Additionally, younger patients can often overcome some of the caregiver barriers (for example, more likely to be able to perform their own driveline dressing changes, etc.) and other social issues that lead to many turndowns based on social factors. However, this is anecdotal speculation. The current series does not currently have the sample size to discriminate at that level of detail.

In regards to race, these findings suggest possible racial bias in selection may lead to the withholding of potentially life-saving therapies. Alternatively, socioeconomic determinants of health may cluster, compounding social and medical issues leading to turndowns. Unfortunately, our study lacks the granularity to further fully evaluate this finding. It is an notable finding of uncertain significance that merits further investigation in the future.

Second, although not statistically significant, our findings do suggest early survival may be higher in patients turned down elsewhere for implantation than in the rest of our cohort. Although seemingly counterintuitive, these findings may simply reflect a very careful assessment of patients declined for implantation elsewhere. Clearly not every patient referred to us for LVAD implantation who was declined elsewhere is ultimately implanted. This remains an important limitation of the present study. Future, prospective evaluation is warranted.

Additionally, there is a trend toward a higher rate of right ventricular failure requiring RVAD in patients turned down for LVAD implantation elsewhere despite similar preoperative acuity. This finding likely reflects an increased surgical/medical risk in this patient population that is difficult to quantify; however, this finding should be carefully considered given the high post-LVAD morbidity and mortality associated with the need for RVAD implantation.^19^

Our finding that social issues represent the majority of LVAD turndowns mirrors the broader patterns seen in assessing LVAD candidacy, where psychosocial factors including family support, caregivers, and medical compliance frequently influence decisions.^11^, ^12^, ^13^ The important question is whether the findings of our study suggest that these social factors are not as important in determining the patient’s overall postoperative trajectory as is broadly assumed; or, whether as a high-volume center, our systems’ established protocols, multidisciplinary assessments, provider expertise, and support staff are somehow able to mitigate the risks associated with these perceived barriers. Broader, multi-institutional studies of these phenomena may help clarify these findings. Additionally, the present study does not address outcomes such as readmission, compliance, LVAD coordinator fatigue, etc. that may be more directly impacted by these social considerations. Further investigation is warranted.

The concept of second opinions or second chances through reevaluation at alternative centers is supported by an emerging literature on the benefits of second opinions in complex cardiac care. Several systematic reviews suggest that patient-initiated second opinions lead to changes in diagnosis and/or treatment in up to 30%-40% of cases, often granting access to newer or innovative therapies that may have been overlooked initially.^20^, ^21^ In cardiac surgery, second opinions have been associated with refined risk stratification and improved patient satisfaction, particularly for higher risk surgery.^22^, ^23^

Furthermore, our center’s status as a high-volume LVAD program likely contributes to our ability to accommodate patients with elevated medical, surgical, and social risks without compromising outcomes. Volume-outcome relationships in LVAD therapy are well-documented, with high-volume centers demonstrated lower mortality rates, reduced adverse events, and increased ability to handle complex cases.^24^, ^25^, ^26^ National databases consistently show that centers performing higher volumes of LVAD procedures benefit from streamlined processes, experienced multidisciplinary teams, and optimized perioperative care protocols, which may mitigate the higher risks associated with the turndown cohort.^18^, ^24^, ^27^ This may explain the comparable results, suggesting that patients turned down for LVAD therapy may benefit from referral to higher-volume centers, potentially improving access to LVAD therapies.^25^, ^28^

In our study, patients turned down for medical or surgical reasons had acceptable and non-inferior short-terms outcomes, suggesting that subjective turndown criteria may not adequately predict real-world outcomes.^11^, ^29^ These findings suggest further research into more standardized selection guidelines and/or earlier referrals to more experienced centers may be warranted. Further studies should explore whether enhanced risk stratification tools of both medical and psychosocial risk could further improve equal access to advanced heart failure therapies, particularly for underrepresented or underserved groups like Black patients, who comprised 46% of our previously turned-down cohort.

### Limitations

There are several limitations to this study. First, as a retrospective study, it may suffer from selection bias and potentially incomplete or inaccurate data extraction. Second, confounding by indication may also exist as patients turned down elsewhere who were ultimately implanted may represent a healthier subset of the overall turndown cohort, inclusive of those not ultimately implanted. Unfortunately, without evaluating this question prospectively, it is impossible to capture all LVAD referrals, including those not implanted. Additionally, as a single-center study, outcomes may reflect institutional expertise rather than broader applicability. Moreover, although we found no differences in short or midterm survival or operative complications, it is possible that long term these patients experience more complications, hospitalizations, or simply require more attention from the LVAD team. These issues could not be evaluated in the present study but merit investigation. Moreover, as a relatively small, single-center study, our study is susceptible to a type 2 statistical error as suggested by the larger database study by Yu et al^30^ where a difference was detected.

Finally, some patients referred for LVAD consideration that were turned down elsewhere were not implanted. Unfortunately, this is difficult to track—some will get palliative care, some will recovery to goal-directed medical therapy, some will get conventional surgery for coronary or valve disease. It is important to emphasize that we are not suggesting that every patient declined for implant is a candidate, merely that some may benefit from a second opinion; therefore, both the patient who is declined and the center who declines should consider this option. Further prospective evaluation is warranted.

In conclusion, patients turned down for LVAD implantation at another center prior to referral to our center had similar short-term morbidity and mortality compared to patients not previously turned down for surgery. These findings suggest that patients with end-stage heart failure who are turned down for LVAD therapy at 1 center should seek or be referred for a second opinion at another center.

## Disclosure statement

All authors have participated in the work and have reviewed and agree with the content of the article. None of the article contents are under consideration for publication in any other journal or have been published in any journal. No portion of the text has been copied from other material in the literature (unless in quotation marks, with citation). I am aware that it is the authors responsibility to obtain permission for any figures or tables reproduced from any prior publications and to cover fully any costs involved. Such permission must be obtained prior to final acceptance.

## Financial support

Drs George, Rawitscher, and Afzal have all served as consultants for Abbott Laboratories, which makes the Heartmate 3 device. The authors have no further relevant financial disclosures.

## Conflicts of Interest statement

The authors declare the following financial interests/personal relationships, which may be considered as potential competing interests: Timothy George reports a relationship with Abbott Laboratories Inc that includes: consulting or advisory. David Rawitscher reports a relationship with Abbott Laboratories Inc that includes: consulting or advisory. Aasim Afzal reports a relationship with Abbott Laboratories Inc that includes: consulting or advisory. If there are other authors, they declare that they have no known competing financial interests or personal relationships that could have appeared to influence the work reported in this paper.
